# Transcriptomic analysis of s-methoprene resistance in the lesser grain borer, *Rhyzopertha dominica*, and evaluation of piperonyl butoxide as a resistance breaker

**DOI:** 10.1186/s12864-020-07354-8

**Published:** 2021-01-20

**Authors:** Maria K. Sakka, Maria Riga, Panagiotis Ioannidis, Georgia V. Baliota, Martha Tselika, Rajeswaran Jagadeesan, Manoj K. Nayak, John Vontas, Christos G. Athanassiou

**Affiliations:** 1 Laboratory of Entomology and Agricultural Zoology, Department of Agriculture Crop Production and Rural Environment, University of Thessaly, Phytokou str., 38446 Nea Ionia, Magnesia Greece; 2 Institute of Molecular Biology & Biotechnology, Foundation for Research & Technology Hellas, 100 N. Plastira Street, GR-70013 Heraklion, Crete Greece; 3 Department of Biology, University of Crete, 70013 Heraklion, Crete Greece; 4 Department of Agriculture and Fisheries, Queensland, Ecosciences Precinct, GPO Box 267, Brisbane, QLD 4001 Australia; 5 Laboratory of Pesticide Science, Department of Crop Science, Agricultural University of Athens, 75 Iera Odos Street, GR-11855 Athens, Greece

**Keywords:** Piperonyl butoxide, S-methoprene, Resistance, Transcriptome analysis, *Rhyzopertha dominica*

## Abstract

**Background:**

The lesser grain borer, *Rhyzopertha dominica* is a serious pest of stored grains. Fumigation and contact insecticides play a major role in managing this pest globally. While insects are developing genetic resistance to chemicals, hormonal analogues such as s-methoprene play a key role in reducing general pest pressure as well as managing pest populations that are resistant to fumigants and neurotoxic contact insecticides. However, resistance to s-methoprene has been reported in *R. dominica* with some reports showing a remarkable high resistance, questioning the use of this compound and other related analogues in grain protection. The current study attempts to identify possible molecular mechanisms that contribute in resistance to s-methoprene in *R. dominica.*

**Results:**

Transcriptome analysis of resistant and susceptible strains of this pest species identified a set of differentially expressed genes related to cytochrome P450s, indicating their potential role in resistance to s-methoprene. Laboratory bioassays were performed with s-methoprene treated wheat grains in presence and absence of piperonyl butoxide (PBO), a cytochrome P450 inhibitor. The results indicate that PBO, when applied alone, at least at the concentration tested here, had no effect on *R. dominica* adult emergence, but has a clear synergistic effect to s-methoprene. The number of produced progeny decreased in presence of the inhibitor, especially in the resistant strain*.* In addition, we also identified CYP complement (CYPome) of *R. dominica*, annotated and analysed phylogenetically, to understand the evolutionary relationships with other species.

**Conclusions:**

The information generated in current study suggest that PBO can effectively be used to break resistance to s-methoprene in *R. dominica*.

**Supplementary Information:**

The online version contains supplementary material available at 10.1186/s12864-020-07354-8.

## Backround

The lesser grain borer, *Rhyzopertha dominica* (F.) (Coleoptera: Bostrychidae) is among the most destructive pests of stored grains, with global distribution [1]. It is a primary feeder and infests a variety of stored products and related commodities [2], which are essential for human nutrition and global food security [1, 3]. Moreover, it is a primary colonizer, thus larvae and adults can easily penetrate the kernels even at low moisture content and complete their life cycle in intact whole grain kernels [2–4]. As a result, most life stages, especially the larvae, are unaffected by contact insecticides that are applied on the external part of the grain kernel [1]. Crucially, *R. dominica* has a rapid population growth resulting in devastating infestation levels, especially at optimal temperatures [1, 5]. Management of *R. dominica* in stored grain and other commodities have been investigated around the globe [1, 6]. In general, its control is currently based on two broad categories of insecticides, the fumigants [7] and contact insecticides [8]. However, it is now well-established that strains of *R. dominica* have developed resistance to both chemical and non-chemical treatments. In particular, high levels of resistance to phosphine [9–11], pirimiphos-methyl [12] and deltamethrin [7, 13] have been reported in many parts of the world, such as Australia, USA and Brazil [9–11]. At the same time, this species cannot be easily controlled by some “traditional” contact insecticides that are applied directly on grains, such as the organophosphorous compound pirimiphos-methyl [12] and the pyrethroid deltamethrin [7, 13]. Moreover, it is well-established that *R. dominica* is less susceptible than other major stored product insect species to non-chemical control methods, such as diatomaceous earths [14], which poses serious challenges to grain industry towards management of this species. Therefore, there is a demand to identify newer, reduced risk compounds that can be effectively used in controlling this important pest.

One of the newer active ingredients that have been registered in many countries for the control of *R. dominica* is the juvenile hormone analogue (JHA), s-methoprene, [15]. JHAs target and disrupt the endocrine system of insects by causing abnormal larval-pupal or nymphal-pupal development and/or even death [16]. In general, s-methoprene has many desirable characteristics, such as good environmental profile and extremely low mammalian toxicity [17, 18] and it is currently considered as a good alternative to many other conventional contact insecticides [15, 19–22]. It also exhibits a considerable residual efficacy on stored grains, thus holding a high potential as a grain protectant for long-term treatment [15, 23].

Although resistance to JHAs is not that frequent, resistance to pyriproxifen in the house fly *Musca domestica* L. (Diptera: Muscidae) and the whitefly *Bemisia tabaci* (Gennadius) (Hemiptera: Aleyrodidae) [24], as well as s-methoprene in mosquitoes [16] have been reported, suggesting that resistance may develop in the case of other species, including *R. dominica*. An s-methoprene resistant strain of *R. dominica* required a very high dose (40 mg kg^− 1^) for its control in wheat grain [25]. This dose rate is approximately 67 times higher than the registered rate applied in Australia, questioning the usage of this insecticide as a grain protectant. Moreover, resistance to s-methoprene may jeopardize the resistance management strategies to phosphine and neurotoxic insecticides [26], on which the inclusion of a JHA, e.g. on a rotation basis, is a key element.

Piperonyl butoxide (PBO), has been used extensively either alone or in combination with other active ingredients as a synergist in crop protection, especially to break resistance to specific group of insecticides such as pyrethroids that exhibits toxicity through mixed function oxidases including CYPs [27]. Several studies reported the interaction of PBO with cytochrome P450s [27, 28]. In the case of stored product protection, PBO has been successfully applied in many different cases [29–31].

The molecular mechanism of s-methoprene resistance has not been fully elucidated yet. In the vinegar fly, *Drosophila melanogaster* Meigen (Diptera: Drosophilidae), the absence of a so-called *methoprene tolerant* (*MET*) gene results in s-methoprene resistance [32, 33]. The protein (MET) encoded by the *MET* gene belongs to the family of basic helix-loop-helix (bHLH)-PAS transcriptional regulators that bind JH with high affinity [34]. MET forms homodimers (Gce in *D. melanogaster* forming heterodimer) in absence of ligand, i.e. Juvenile hormone III (JH-III), the growth juvenile hormone synthesized in most insects, or a synthetic mimic. In presence of either ligand, MET homodimer dissociates and their presence leads to dissociation of the MET dimer and thus binding with the ligand (JH-III or synthetic mimic). Ligand binding and immunoprecipitation assays where both MET monomers carry the V297F mutation, indicated resistance to s-methoprene thus they were not dissociated compared to the wild type counterpart [34]. Further experiments indicated that methoprene binds to PAS-B domain of the MET protein. Also, functional assays by knocking down *MET* in *T. castaneum,* render the insects resistant to the natural JH and as well as s-methoprene [35]. Alternatively, resistance to s-methoprene in other species has been associated with high activity of P450 monooxygenases and esterases, which probably also contribute to resistance to s-methoprene and other JHAs [36, 37]. However, detailed research revealing the exact relationship between s-methoprene and CYPs is not established, but it has been shown that P450s can metabolize JHAs, as in the case of pyriproxifen [38], which consists an indication that the same phenomenon may occur in the case of s-methoprene.

Resistance to s-methoprene has not been analysed yet in *R. dominica*, largely due to the lack of genomic resources for this pest species. RNA sequencing technologies have evolved rapidly in the last years [39]. They allow the study of transcriptomes without necessarily relying on a reference genome, thus greatly facilitating the study of several non-model species. Subsequently, comparison of gene transcription levels between insecticide resistant and insecticide-susceptible insect strains can lead to candidate genes that could play a role in the observed resistant phenotype. Such analysis has been performed in several insects and mites [40–43], providing not only a better understanding of insecticide resistance, but also valuable genomic resources that prove useful for studying different aspects of the biology of arthropods that constitute the most diverse animal clade [44–46].

In this regard, the aim of the present work was to investigate, for the first time, the mechanisms underlying s-methoprene resistance in *R. dominica.* We used s-methoprene-resistant and susceptible strains and compared their response to s-methoprene alone, but also in combination with PBO and mortality and progeny production were measured. The bioassays showed that the combined use of s-methoprene + PBO increased the efficacy of the former, thereby suggesting a possible involvement of CYPs in the resistance mechanism. Subsequently, we sequenced the transcriptomes of s-methoprene-resistant and susceptible strains and identified the Cytochrome P450 (CYP) genes. Interestingly, their analysis revealed that a number of them were significantly up-regulated in the resistant strain and are thus worth of further investigation to determine their role in insecticide resistance to JHAs.

## Results

### Laboratory bioassays

**Fig. 1:**
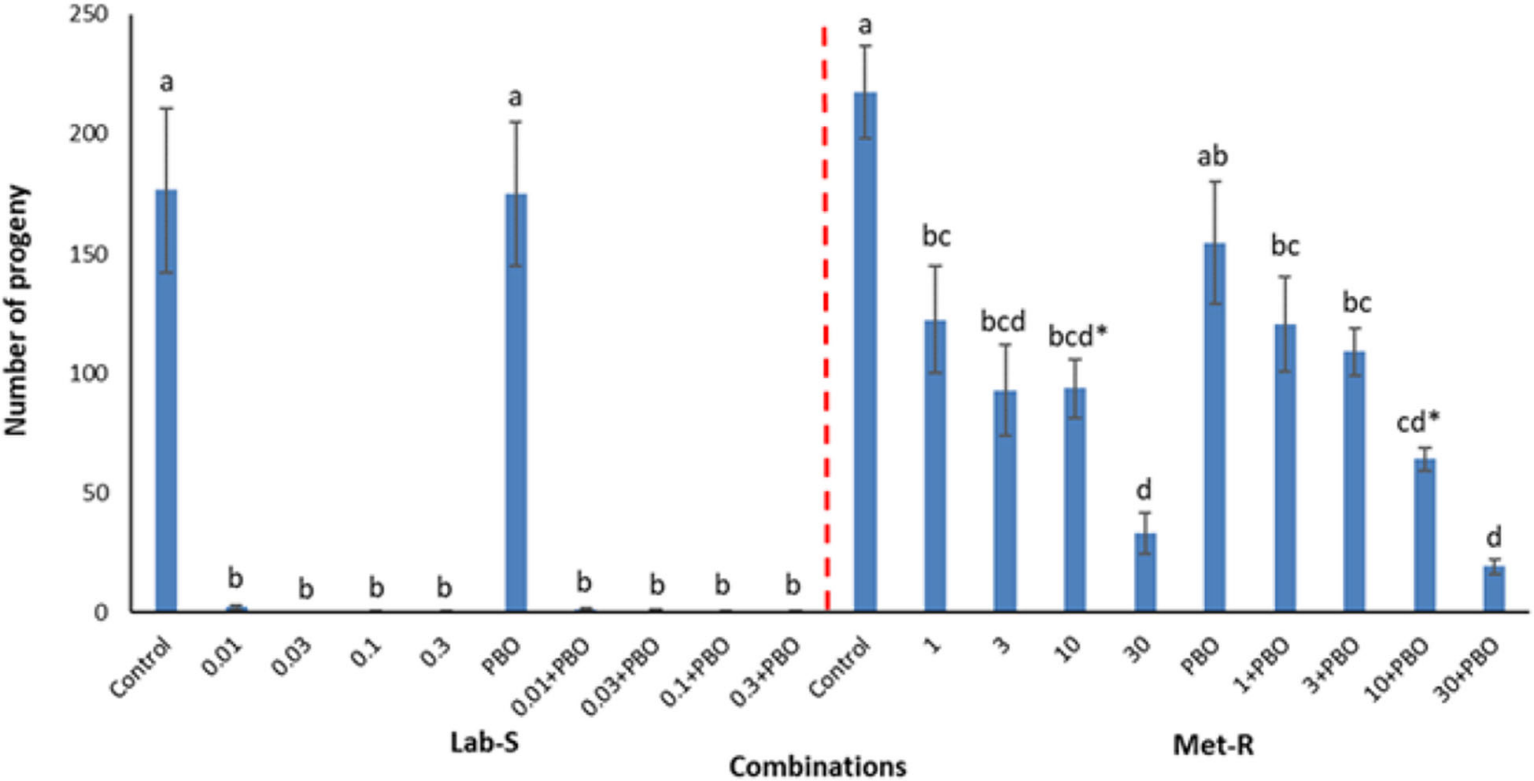
Mean number (±SEM) of *Rhyzopertha dominica* progeny production (expressed as adults/vial) for the susceptible (Lab-S) and resistant (Met-R) strain for all the combinations tested (control, 0.01 mg/kg, 0.03 mg/kg, 0.1 mg/kg, 0.3 mg/kg, PBO, 0.01 mg/kg + PBO, 0.03 mg/kg + PBO, 0.1 mg/kg + PBO, 0.3 mg/kg + PBO for susceptible and control, 1 mg/kg, 3 mg/kg, 10 mg/kg, 30 mg/kg, PBO, 1 mg/kg + PBO, 3 mg/kg + PBO, 10 mg/kg + PBO, 30 mg/kg + PBO for resistant). Within each bar and strain, means followed by the same lowercase letter do not differ significantly according to Tukey Kramer HSD test at *P*< 0.05. Where no letter exist, no significant differences were noted. Means with asterisk (*) for the application with s-methoprene alone are significantly different for the respective mean of the combination with s-methoprene and PBO at the resistant strain (Met-R)

Treatment effects were significant (Table 1). Parental mortality was low for 7, 14 and 21 days for both strains. Parental mortality for the control Lab-S was 0.1 and 12% for the Met-R. Moreover, for Lab-S and Met-R the lowest parental mortality was 6.7 and 2.2 and the highest 26.7 and 17.8 respectively (Additional file 1: Fig. S1). Regarding progeny production counts, adult emergence was generally higher in the case of the resistant strain, as compared to the respective figures of the susceptible strain, even in the untreated grains (Fig. 1). Moreover, the application of PBO alone, for both strains, had no effect, as the numbers of adults that had been emerged after the termination of the incubation period were extremely high (> 150 adults/vial), and comparable to those in the controls (Fig. 1). Still, for the resistant strain, the application of PBO alone caused a slight reduction in progeny production, in comparison with the control vials. In the case of the Lab-S strain, the combination of PBO with s-methoprene gave similar results with the application of s-methoprene alone. For this strain, when s-methoprene was applied either alone or in combination, progeny production was generally higher at 0.01 mg/kg than that for the other concentrations. Nevertheless, for the susceptible strain, progeny production ranged between 0.2 and 2.3 adults/vial (Fig. 1). In contrast, for the resistant strain, when s-methoprene was applied alone, progeny production was significantly lower than that in the control vials (Fig. 1). However, there was a considerably high offspring emergence, regardless of the concentration. The increase of the concentration from 1 to 30 mg/kg resulted in a gradual decrease on the number of emerged *R. dominica* adults, from 122 to 33 individuals/vial. Similarly, when s-methoprene was applied with PBO, the increase in the concentrations reduced progeny production from 120 to 19 individuals/vial (Fig. 1). Furthermore, for the two lowest s-methoprene concentrations, progeny production was not affected, regardless of the presence of PBO. Nevertheless, for the two higher concentrations, progeny production of *R. dominica* was considerably lower when s-methoprene was applied in combination with PBO, than for the application of s-methoprene alone (Fig. 1).

**Table 1:** ANOVA parameters for progeny production of *R. dominica* susceptible (Lab-S) and resistant strain (Met-R) (error *df*=80)

Source	*df*	Susceptible		Resistant	
		F	P	F	P
Whole Model	9	25.9	< 0.001	12.6	< 0.001
Intercept	1	61.2	< 0.001	399.2	< 0.001
Treatment	9	25.9	< 0.001	12.6	< 0.001

### Transcriptome sequencing

In order to better study the molecular basis of the observed resistance, the transcriptome of *R. dominica* was sequenced, yielding a total of > 688 million Illumina reads. These reads were then assembled de novo with Trinity since there is no available reference genome sequence. The assembled transcriptome contained a total of 117,265 putative transcripts (Table 2). The quality of the assembly is very good, as evidenced by the BUSCO analysis [47], which showed that > 98% of the conserved insect genes are present in the assembly (Table 3).

**Table 2:** Transcriptome assembly summary

**Number of transcripts**	**117,265**
**Number of unigenes**	**64,209**
**Predicted peptides**	**45,255**
**with a BLAST hit vs Uniref50, e-value < 10**^**− 5**^	**42,123**
**against Metazoa**	**38,856**
**against Arthropoda**	**34,272**
**against Coleoptera**	**23,119**
**against Bacteria**	**189**
**with an InterPro domain (from InterProScan)**	**35,673**
**with an assigned GO term (from InterProScan)**	**26,482**
**with a Pfam domain (from InterProScan)**	**32,965**
**BUSCO quality assessment**	
**Number of complete Insecta BUSCOs**	**1594 (96.2%)**
**Number of fragmented Insecta BUSCOs**	**21 (1.3%)**
**Number of Insecta BUSCOs not found**	**43 (2.5%)**

**Table 3:** Detailed RNA sequencing results for each *R. dominica* strain

Sample	Total bp	Read count	GC (%)	Q20 (%)	Q30 (%)
**Met-R_A**	6,917,873,598	68,493,798	**44.98**	**97.37**	**92.63**
**Met-R_B**	8,078,960,912	79,989,712	**46.22**	**97.69**	**93.27**
**Met-R_C**	6,910,050,744	68,416,344	**46.81**	**97.59**	**93.12**
**Lab-S_A**	8,238,534,852	81,569,652	**45.99**	**97.33**	**92.54**
**lab-S_B**	8,092,498,346	80,123,746	**46.38**	**97.73**	**93.46**
**Lab-S_C**	7,920,684,014	78,422,614	**46.84**	**97.25**	**92.41**

After calculating transcript abundance a Principal Component Analysis (PCA) was run in order to verify the quality of the biological replicates. It is evident that the replicates of the same strain clustered together, but also are separated from the replicates of the other strain (Additional file 2: Fig. S2). These results show that the sequencing data are of good quality and can be used in downstream analyses.

### Investigating target site-mediated resistance

The sequence polymorphism analysis as well as expression levels in the *MET* gene between the Lab-S and Met-R *R. dominica* strains did not detect any significant differential expression. However, examination of the open reading frame (ORF) of *MET* between the two strains revealed the occurrence of a non-synonymous amino acid substitution at position 489 of the amino-acid sequence in the Met-R strain. The observed substitution leads to the replacement of a Pro by Leu. However, this mutation is not fixed in Met-R, it is present in only 33% of the reads, and, finally, is located outside of the PAS-B conserved domain.

### Investigation of non-target site resistance mechanisms based on differential expression and qPCR validation

**Fig. 2:**
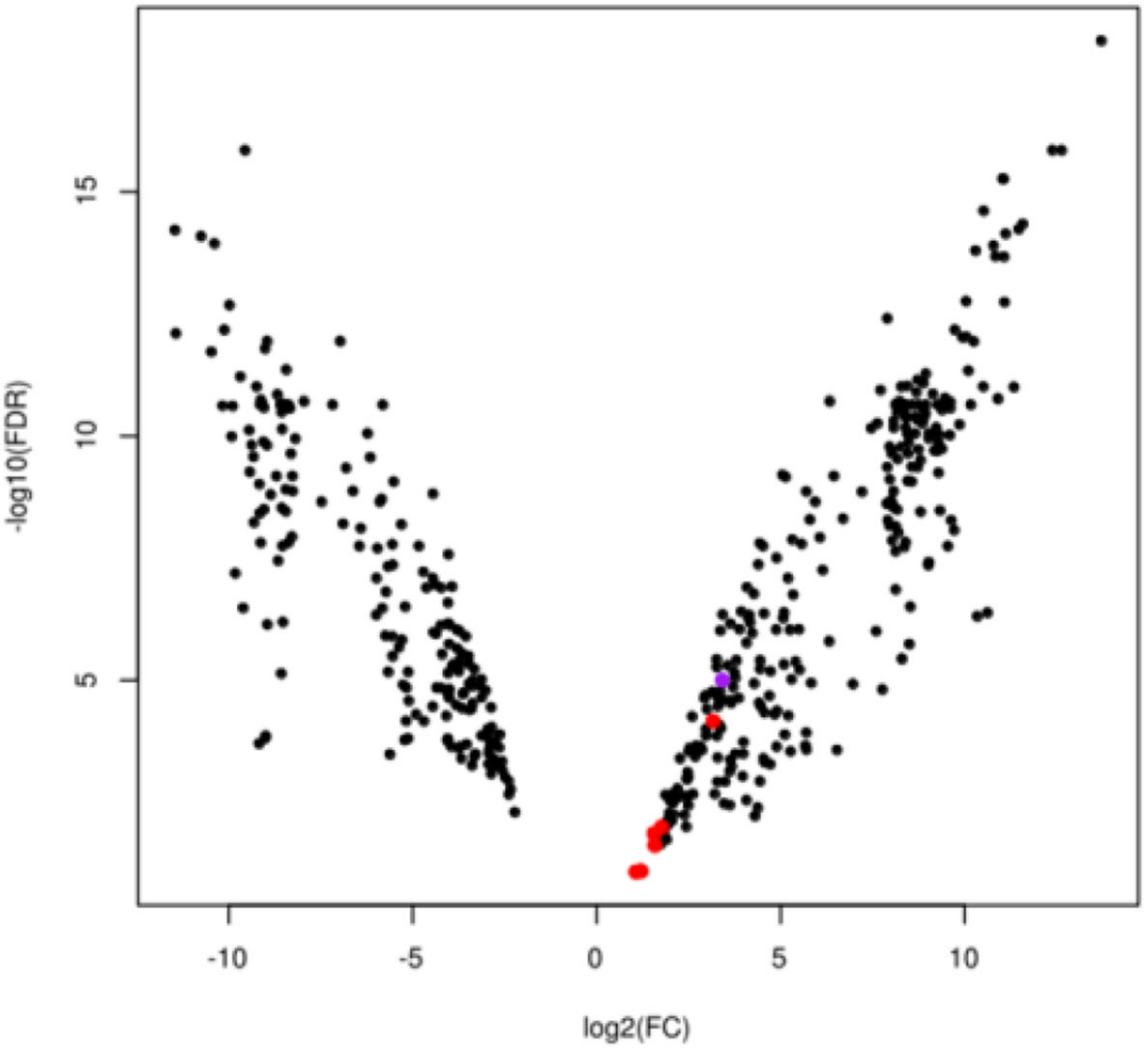
Overview of the differentially expressed (|log_2_FC| > 2 and also *p*-value < 0.001) genes between the resistant and the susceptible to s-methoprene strains of *R. dominica*. In total, there were 465 differentially expressed unigenes, of which 276 are up-regulated in the resistant strain, whereas the remaining 190 are up-regulated in the susceptible strain. The data points corresponding to P450s have been colored as red, whereas the one corresponding to the UGT is colored as purple

Differential expression (DE) analysis was done on all the 117,265 assembled transcripts, at the unigene level. This analysis showed that 275 unigenes were up-regulated in the Met-R strain compared to Lab-S, whereas another 190 were down-regulated (Fig. 2). No significantly over-represented GO terms or KEGG pathways were found in either the up-or down-regulated set of genes (p_adj_ < 0.01).

**Fig. 3:**
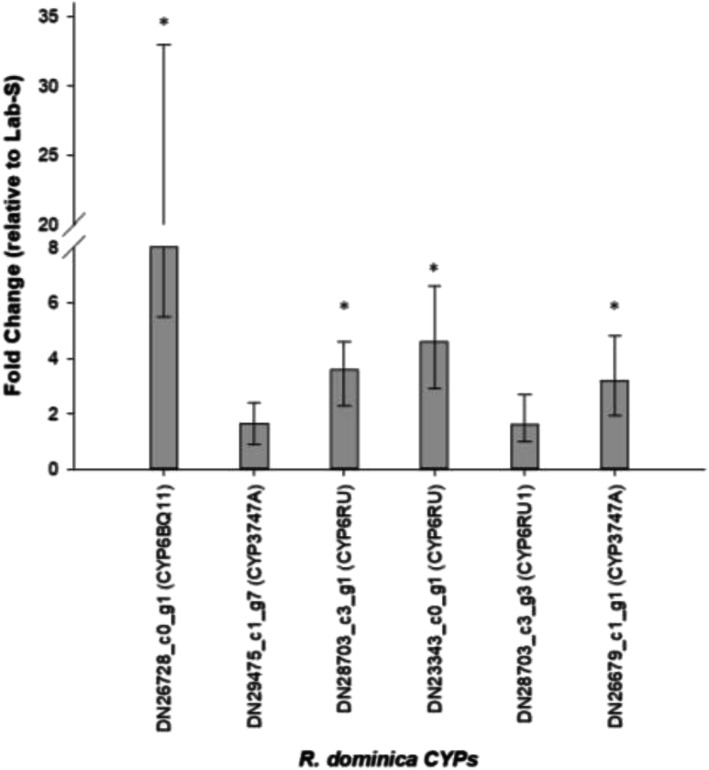
Relative expression levels of the six CYPs. Expression levels are depicted relative to Lab-S reference susceptible strains. Error bars represent 95% confidence intervals. Asterisks indicate significantly different expression (p-value < 0.05)

Interestingly, we identified a number of up or down-regulated unigenes that have a similarity to detoxification enzymes (Table 5). These include six CYPs (DN26728_c0_g1, DN29475_c1_g7, DN28703_c3_g1, DN23343_c0_g1, DN28703_c3_g3, DN26679_c1_g1), one glutathione S-transferase (GST) (TRINITY_DN20738_c0_g1), and one UDP-glucosyltransferase (UGT) (DN28972_c1_g2). The CYPs as well as the UGT were up-regulated in the Met-R strain, whereas the GST was up-regulated in the Lab-S strain. The difference in expression levels was statistically significant for all these unigenes (FDR < 0.05). The over-expression levels of the identified CYPs were validated by qPCR with CYP6BQ11 (DN26728_c0_g1), CYP6RU (DN28703_c3_g1 and DN23 343_c0_g1) and CYP3747A (DN26679_c1_g1) displaying significant (*p*=value < 0.05) up-regulation of > 10-, 4- and 3-fold in the Met-R strain, compared to the Lab-S strain (Fig. 3).

### Detailed study of putative CYPs

**Fig. 4:**
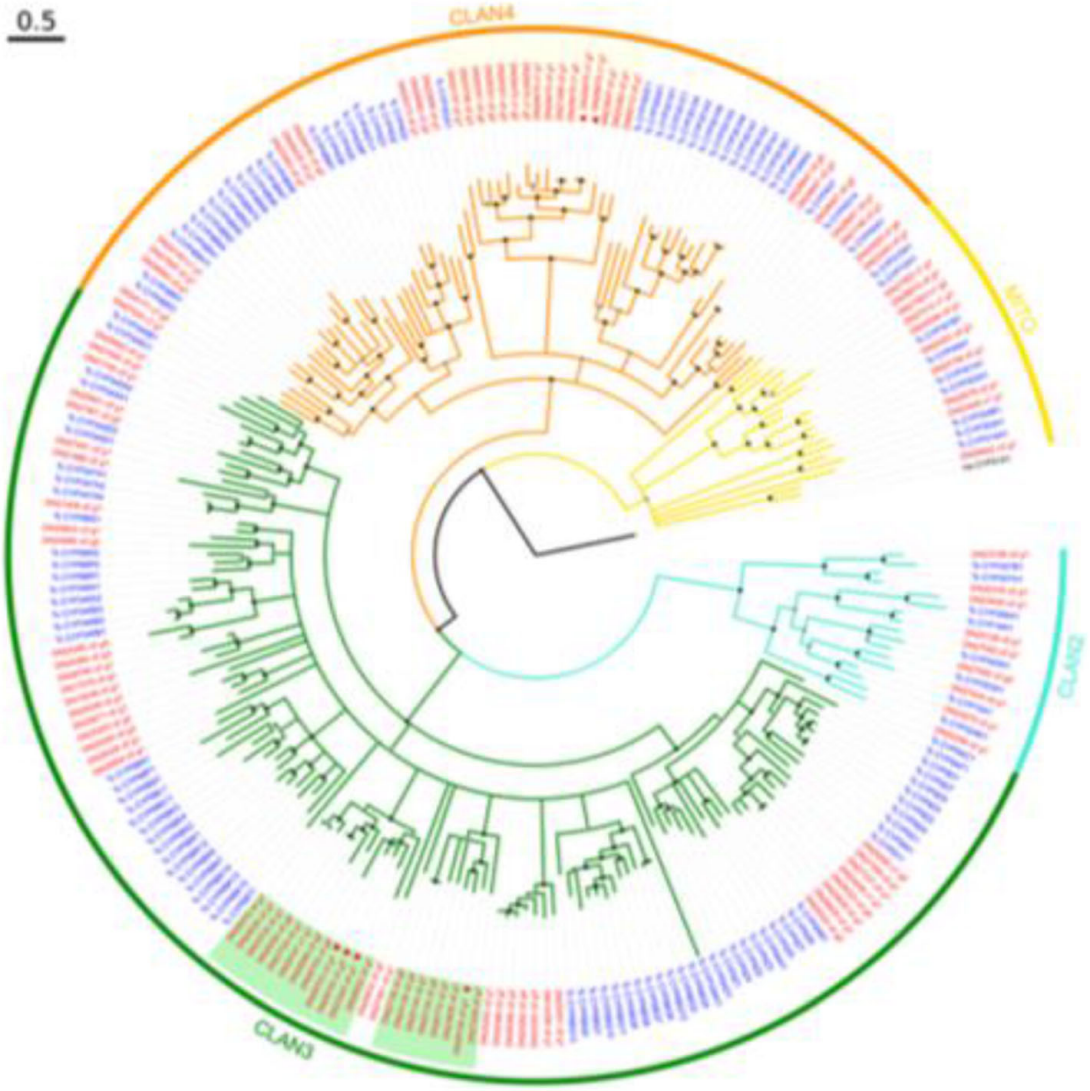
Phylogenetic analysis of the CYP genes identified in *R. dominica*. This analysis showed that all identified *R. dominica* CYPs could be classified into one of the known *T. castaneum* clans. Furthermore, the differentially expressed CYPs belong to Clan 3 (four unigenes) and Clan 4 (two unigenes). All *R. dominica* genes were classified into one of the four known CYP clans previously found in the beetle *T. castaneum*. Bootstrap values > 75% are represented as black dots, whereas nodes with bootstrap support between 50 and 75% are shown as grey dots. Nodes with bootstrap support < 50% are collapsed. The *R. dominica*-specific expansions in Clans 3 and 4 containing the up-regulated CYPs are highlighted in light orange and light green, respectively. CYPs whose log_2_FC is > 2 are marked with a red asterisk, whereas those with a log_2_FC between 1 and 2 are marked with a red triangle. The scale bar is in substitutions per site

*Rhyzopertha dominica* transcripts containing the InterPro domain IPR001128, were searched and annotated as putative CYPs or CYP fragments. The analysis revealed 396 probable CYP isoforms of *R. dominica* putatively originating from 111 unigenes. Maximum Likelihood phylogenetic analysis was performed on the largest isoform from each unigene, using the *T. castaneum* CYP genes [48] as a reference. All the *R. dominica* CYPs were classified into one of the four known CYP clans existing in *T. castaneum* (Fig. 4, Table 4). Furthermore, this analysis revealed at least eight *R. dominica*-specific clades in Clans 3 and 4 for some of which a clear classification within the respective clade was not possible. In addition, the phylogenetic analysis also shows that there are four different unigenes in *R. dominica* that cluster with the *T. castaneum* CYP12H1. This is an indication of probable duplication events that led to multiple copies of this CYP gene in *R. dominica*.

**Table 4:** Summary of the phylogenetic analysis of *R. dominica* P450s

Clan	***R. dominica*** unigenes^a^	***T. castaneum*** genes
Clan 2	9	8
Clan 3	58	68
Clan 4	33	40
Mitochondrial	11	9
**Total**	**111**	**125**

^a^Classification was done using a threshold of > 50% bootstrap support

Interestingly, two of the identified CYPs were significantly up-regulated (FDR < 0.001, log_2_FC > 2) in the Met-R strain. Another four also appear to be significantly up-regulated, albeit at a lower degree (FDR < 0.05, log_2_FC > 1.44). Four of the six CYPs belong to Clan 3, whereas the other two belong to Clan 4 (Fig. 3, Table 5). A more precise placement of these clades was not possible due to the low bootstrap support values (< 50%) of the respective branches. Nevertheless, expert manual curation by Dr. David Nelson annotated these genes as similar to the genes CYP6BQ11, CYP3747A (from *Oryctes borboronicus*), CYP6RU (from *Photinus pyralis*), and CYP6RU1 (from *Photinus pyralis*) (Table 5; Additional file 3: Table S1).

**Table 5:** Up-regulated detoxification enzymes in the Met-R strain

Gene family	Unigene ID	Phylogenetic classification	Best Uniref50 match	BLAST-based annotation	Expert annotation^a^	log_2_FC	FDR
P450	DN26728_c0_g1	Clan 3	M4W605	CYP6BQ8	CYP6BQ11	3.56	8.9e-12
	DN29475_c1_g7	Clan 4	A0A0T6B959	–	CYP3747A	2.12	1.6e-04
	DN28703_c3_g1	Clan 3	A0A0T6BCU6	–	CYP6RU	1.96	2.3e-04
	DN23343_c0_g1	Clan 3	V5GHG9	CYP6A1	CYP6RU	1.96	1.5e-03
	DN28703_c3_g3	Clan 3	D7EJA5	CYP6BK4	CYP6RU1	1.62	1.6e-03
	DN26679_c1_g1	Clan 4	N6URV7	CYP349B1	CYP3747A	1.44	3.6e-02
UGT	DN28972_c1_g2	N/A	A0A1Y1JU93	UGT	N/A	3.87	2.3e-02

^a^Expert manual annotation was kindly provided by Dr. David Nelson

## Discussion

Τhe frequency of cases of insecticide resistance of insects infesting stored products has been increased in the last decades [12, 26, 49–51]. S-methoprene is an insect growth regulator which plays a pivotal role in mitigating resistance to several contact insecticides and fumigants [15, 52]. Although it has a unique mode of action, and it has not been previously used as grain protectant, there are reports of high levels of resistance to s-methoprene in *R. dominica* [24, 53], which may question its use in the near future [26]. While many studies have focused on the phenotypic characterization of resistance, the current work elucidates molecular mechanisms of resistance to s-methoprene in *R. dominica*, with a perspective of developing suitable resistance management practices.

Our study clearly indicated that the simultaneous application of s-methoprene + PBO, increased the insecticidal effect of s-methoprene (Fig. 1) and all the above clearly indicate that PBO, which when applied alone, at least at the concentration tested here, had no effect on *R. dominica* adult emergence, but has a clear synergistic effect to s-methoprene.

The use of PBO as a synergist has been extensively used in stored product protection, but most of the studies available are about pyrethroids. For instance, the application of PBO with natural pyrethrum were found to increase the efficacy of diatomaceous earths for the control of *R. dominica* on different grains [31]. Similar results have been reported for the application of natural pyrethrum alone [54]. Deltamethrin resistance has been shown to reduce from 223-fold to 21-fold using the CYP inhibitor PBO against a pyrethroid resistant population of the cotton armyworm, *Helicoverpa armigera* (Hübner) (Lepidoptera: Noctuidae) [55]. In the case of stored product insects, the granary weevil, *Sitophilus granarius* (L.) (Coleoptera: Curculionidae) was tested with PBO and fenitrothion and it was found that there is a positive synergism between them [56]. Our results suggest that this combination can also be effective in the case of resistance to JHA by stored product insects, but, to our knowledge such an approach has not been implemented yet.

Sequence analysis of the *MET* gene identified a P489L substitution in the resistant Met-R strain, but not in the susceptible Lab-S. A mutational change at position 297 in the MET protein was reported earlier in s-methoprene-resistant *T. castaneum* that has explicitly exhibited reduced binding affinity to s-methoprene [34]. However, the herein identified P489L mutation is located at the C-terminus of the gene and outside of the PAS-B domain that has been previously implicated in ligand binding. The functional role of P489L and its contribution to resistance remains to be investigated.

Our analysis identified a total of 111 *R. dominica* CYPs, a number relatively close to the 143 CYPs identified in the closely related *T. castaneum* [48]. The missing genes in *R. dominica* are most probably due to the fact that not all the CYP genes were transcribed at the samples we sequenced. Six of these CYPs were up-regulated in the resistant Met-R strain (Fig. 2), with four of them belonging to Clan 3 and the other two to Clan 4. However, their comparison to *T. castaneum* CYPs showed that they cannot be reliably grouped to any family within these clans (Fig. 3). Evidence supports that CYPs metabolize JHAs, such as in the case of pyriproxifen [38]. Additionally, heterologously expressed CYPs from *A. gambiae* were shown to be capable of metabolizing pyriproxifen, with CYP6P3 to be the strongest metabolizer [57]. Moreover, microsomal CYPs are capable of metabolizing s-methoprene when incubated with housefly microsomes [58]. Application of sub-lethal concentrations of s-methoprene in Sf9 cells and the fall armyworm indicated induction of the expression of CYPs in Sf9 cells, most of which belong to the CYP9 family, whereas in live insects CYP9A28 was differentially expressed in response to s-methoprene [59]. Thus, CYPs could be potentially involved in s-methoprene resistance and are therefore worth of a more detailed analysis. To this end, and in order to be able to properly classify each of the identified *R. dominica* CYPs, we conducted a Maximum Likelihood phylogenetic analysis with the manually curated set of CYPs of *T. castaneum* [48]. The results of this analysis showed that all six up-regulated CYPs belong to three well-supported clades which, nevertheless, only contained *R. dominica* genes (Fig. 3). This, of course, did not allow for a more precise placement of these clades within the respective CYP clans. Nevertheless, expert manual annotation by Dr. David Nelson assigned specific functions to each one of them, thus providing hints for their possible function (Additional file 4: Table S2).

More specifically, CYPs belonging to the CYP6 family (Clan 3) have been characterized and shown to metabolize xenobiotics and plant allelochemicals in several insect species, such as *M. domestica*, the African malaria mosquito, *Anopheles gambiae* Giles (Diptera: Culicidae) and others [60]. However, there was no detection of a specific CYP gene capable of metabolizing s-methoprene. For example, the *M. domestica* CYP6A1 was not able to metabolize s-methoprene and hydroprene, while other JHs such as juvenile hormones I and III were metabolized [61]. Nevertheless, transcriptomic analysis of a pyriproxyfen (another JHA insecticide)-resistant strain of the greenhouse whitefly, *Trialeurodes vaporariorum* Westwood (Hemiptera: Aleurodidae) suggested that the most highly up-regulated CYPs (log_2_FC between 2.68 and 2.91) also belonged to the CYP6 family [62], but qPCR analysis indicated that a CYP belonging to the CYP4 family (Clan 4) is highly upregulated in the pyriproxyfen selected strain. Here, the expression levels of 6 CYPs from *R. dominica* were validated by qPCR indicating two of them to be significantly over-expressed in the Met-R strain in comparison to the Lab-S. Given the synergistic effect of PBO that argues in favor of a P450-mediated resistance and validated over-expression of four CYPs, point towards their functional expression and characterization which will give evidence for their role in metabolic activity and implication in the observed resistance.

## Conclusions

The results of the present study indicate that resistance to s-methoprene is potentially mediated by cytochrome CYPs. Moreover, our bioassay data suggested that the simultaneous application of PBO and s-methoprene can be used with success to mitigate resistance to s-methoprene in *R. dominica*. In order to investigate whether CYPs are involved in resistance we sequenced a susceptible (Lab-S) and a resistant (Met-R) to s-methoprene strain of *R. dominica*, using RNA-seq. Data analysis identified a number of up-regulated genes that bear significant similarity with CYPs and could thus be involved in the detoxification of s-methoprene. Most importantly, the herein generated transcriptome assembly is the only genomic resource for *R. dominica* and it can serve as a reference in future projects studying the biology of this important pest. Additionally, our results suggest that PBO acts as a “resistance breaker” and should therefore be considered towards the direction of resistance management. This is particularly important in the case of s-methoprene, as it is classified among the insecticides with the lowest mammalian toxicity that are currently in use as grain protectants.

## Methods

### Reagents and chemicals

The pure analytical grade chemicals of s-methoprene and PBO-8 were obtained from Dow Agrosciences Ltd. (CPC2 Capital Park, Fulbourn, Cambridge, England, CB21 5XE). In addition, the commercial formulations of these two chemicals, such as Diacon IGR^TR^ and PBO8-Synergist, containing 33.6% active ingredient (a.i) of s-methoprene and 91.3% of PBO, respectively, were used for the tests.

### Insect strains

Two strains of *R. dominica*, which are susceptible (Lab-S) and resistant to s-methoprene (Met-R), respectively, were used in this study. The Lab-S was collected form a grain storage shed at Oakey in 1971, whereas, Met-R strain was collected from Roma, Queensland [26]. Since then, the insect populations of these two strains were reared on whole wheat kernels and maintained at standard room temperature and relative humidity (RH) at Queensland Department of Agriculture and Fisheries (QDAF), Australia. Adult beetles less than three weeks old were randomly selected from the culturing jars and used in the bioassays.

#### Laboratory bioassays

Two sets of s-methoprene concentrations were used in the bioassays. These include, 0, 0.01, 0.03, 0.1 and 0.3 mg kg^− 1^ for Lab-S and 0, 1, 3, 10 and 30 mg kg^− 1^ for Met-R. For PBO bioassay, the wheat grains were applied with a recommended label rate for combinations, 0.013 lt per 45.3 kg of wheat. Untreated clean and infestation-free wheat grains were used for treatments. The moisture content of the grain was adjusted to 13.5% before the initiation of the experiment. Three lots of wheat containing 2 kg each were sprayed with different dose rates of s-methoprene alone, PBO alone and the combination of s-methoprene + PBO; hence, the combinations of the formulations were: a) s-methoprene alone, b) PBO alone and c) s-methoprene with PBO, in all possible combinations (control, 0.01 mg/kg, 0.03 mg/kg, 0.1 mg/kg, 0.3 mg/kg, PBO, 0.01 mg/kg + PBO, 0.03 mg/kg + PBO, 0.1 mg/kg + PBO, 0.3 mg/kg + PBO for Lab-S and control, 1 mg/kg, 3 mg/kg, 10 mg/kg, 30 mg/kg, PBO, 1 mg/kg + PBO, 3 mg/kg + PBO, 10 mg/kg + PBO, 30 mg/kg + PBO for Met-R). The required volume of each treatment including the combinations, was applied using a specialized airbrush (Badger 100, Kyoto BD-183 K Grapho-tech, Japan). An additional Series of 2 kg wheat lots, sprayed with water in parallel to each treatment, was used as untreated control. Twenty grams (20 g) of grain samples from each treatment was selected for bioassays. This sample was placed inside cylindrical bioassay vials (3 cm in diameter, 8 cm in height). Ten adults of *R. dominica* were released into each vial. The vials were then placed in incubators set at 27.5 °C and 75% RH. The mortality of adults was recorded after 7, 14 and 21 days of exposure. Thereafter, all parental adults were removed from the vials, and the vials with the treated grain were returned to the same incubators and maintained for 65 d more to ensure that the immatures in the treated grain will develop up to the adult stage. Then, the number of adults that emerged in treatments and control were compared and per cent reduction in progeny production was estimated. The entire experiment was repeated three times (jars) with each containing 3 subreplicates (vials).

### RNA isolation, library construction sequencing and qPCR validation

Ten 2nd to 3rd instar larvae of *R. dominica* of Lab-S and Met-R strains were pooled respectively and preserved in RNA later, and total RNAs of each was extracted using the GeneJet RNA Purification kit (ThermoScientific), according to the manufacturer’s protocol. The extracted RNA was treated with Turbo DNase (Ambion), in order to remove any traces of genomic DNA. The purity and concentration of RNA were estimated using a Nanodrop spectrophotometer based on 260/280 and 260/230. RNA samples were sent to Macrogen (Korea) for mRNA paired-end library construction with the Illumina Truseq stranded mRNA sample preparation kit, following the manufacturer’s instructions. Each library was sequenced with the paired-end method for a read length of 100 bp. Two μg of RNA was used for cDNA synthesis using the reverse transcriptase kit from Minotech (Heraklion, Greece), according to the manufacturer’s instructions. qPCR validation was conducted for a subset of genes. The primers used are shown on Additional file 6: Table S4. Briefly, a 5-fold dilution series of pooled cDNA was used to assess the efficiency of the qPCR reaction for each gene-specific primer pair. A no template control (NTC) was also included to detect possible contamination. The reactions consisted of 0.6 μM primers each, and Kapa SYBR FAST qPCR Master Mix (Kapa-Biosystems). Experiments were performed using 3 biological and 3 technical replicates for each gene. The levels of the validated genes were measured by Real-time qPCR (RT-qPCR) amplification on a CFXConnect (BioRad). Relative expression levels were calculated as previously described [63].

### Computational analyses

RNAseq reads from both strains (total of ~ 688 million reads) were assembled with Trinity v2.5.1 [64], using parameters “--seqType fq --SS_lib_type RF --max_memory 350G --CPU 24”. InterProScan v5.28–67 [65] was used in order to identify conserved domains within each assembled transcript. Moreover, BLAST v2.8.0+ [66] searches were run in order to identify similarities using the Uniref50 database that is specifically built for similarity-based functional annotation [67].

Transcript abundance was estimated with Kallisto [68]. Next, the scripts bundled with Trinity were used for running the differential expression analysis with EdgeR [69] in order to find transcripts that were differentially expressed between the resistant and the susceptible strain (FDR < 0.05). Custom Perl and bash scripts were used for parsing the EdgeR output and identifying genes of interest. Gene Ontology (GO) term analyses were done using gProfiler [70].

For the detection of polymorphisms in the *methoprene tolerant* gene we firstly mapped the raw reads to the Trinity transcripts using hisat2 [71], then generated a mpileup file with samtools [72], and searched for SNPs with VarScan v2.4.4 [73]. Finally, the identified SNPs were visually inspected across the extracted methoprene tolerant transcript using samtools and the data were loaded into the Integrative Genomics Viewer v2.6.3 [74].

In order to identify transcripts with similarity to cytochrome P450 (CYP) genes we first ran the TransDecoder program that is bundled with Trinity v2.8.5 [64] and obtained the encoded peptides in each transcript. Subsequently, putative CYP-related proteins were identified by the presence of the IPR001128 InterPro domain, in the InterProScan output file. The curated CYPs set identified in *T. castaneum* were obtained from [48] and used as a reference for classifying the herein identified *R. dominica* CYPs. Finally, the early-diverged CYP51A1 [75] from *Homo sapiens* was used as an outgroup. Multiple sequence alignment (Additional file 5: Table S3) was performed with MAFFT v7.271 [76] with parameters “--auto --threads 8” and trimming was done with Trimal v1.2rev59 [77], with parameters “--gt 0.50”. A Maximum Likelihood phylogeny with 100 bootstrap replicates was inferred with RAxML v8.2.11 [78], with parameters “-m PROTGAMMAAUTO”. Branches with < 50% bootstrap support were collapsed with TreeGraph2 [79] and the resulting Newick tree was loaded to a locally deployed instance of EvolView v2 [80] for post-processing. The vector graphics editor Inkscape v0.92 was used for the final polishing.

### Bioassay data analysis

The data of progeny production were analyzed separately for each strain using ANOVA to test the treatment effects. When preliminary tests indicated that variances were not equal, the data were transformed to log (x+ 1) (for the susceptible strain O’Brien test: *F*=1.01, *P*=0.437; for the resistant strain O’Brien test: *F*=1.84, *P*=0.073). Means were separated by using the Tukey-Kramer HSD test at the 5% level. For each strain the Student’s t-test was used to determine differences between s-methoprene alone and s-methoprene with PBO. Statistical analysis was performed by using the JMP 7 software (SAS Institute Inc., Cary, NC, USA).

## Supplementary Information

**Additional file 1: Fig. S1.** Mean mortality (±SEM) of *Rhyzopertha dominica* after 7, 14 and 21 days of exposure for the susceptible (Lab-S) and resistant (Met-R) strain for all the combinations tested (control, 0.01 mg/kg, 0.03 mg/kg, 0.1 mg/kg, 0.3 mg/kg, PBO, 0.01 mg/kg + PBO, 0.03 mg/kg + PBO, 0.1 mg/kg + PBO, 0.3 mg/kg + PBO for susceptible and control, 1 mg/kg, 3 mg/kg, 10 mg/kg, 30 mg/kg, PBO, 1 mg/kg + PBO, 3 mg/kg + PBO, 10 mg/kg + PBO, 30 mg/kg + PBO for resistant).

**Additional file 2: Fig. S2.** Principal components analysis of the transcript expression levels between the resistant (Met-R - blue) and susceptible (Lab-S - red) strains. The two strains are clearly different from each other, a prerequisite for downstream analyses.

**Additional file 3: Table S1.** Corresponding information regarding sequence identity of CYPs.

**Additional file 4: Table S2.** Details of all up- and down-regulated genes between the resistant Met-R and the susceptible Lab-S strains (p_adj_ < 0.001, log_2_|FC| > 2).

**Additional file 5: Table S3.** Amino acid sequences used in the phylogenetic study of the *R. dominica* P450s, presented in Fig. 4.

**Additional file 6: Table S4.** Primers used for the validation qPCRs.

## Data Availability

The sequencing reads are available from the Sequence Read Archive (SRA) under the bioproject accession PRJNA605183. Additional data and custom scripts are available upon request.

## Abbreviations

PBO: Piperonyl butoxide
JHA: Juvenile hormone analogue
DE: Differential expression
