# Supplementary figures and images for: Transcriptomic analysis of s-methoprene resistance in the lesser grain borer, Rhyzopertha dominica, and evaluation of piperonyl butoxide as a resistance breaker
Source: BMC Genomics. 2021 Jan 20;22:65. doi: 10.1186/s12864-020-07354-8 (PMC7819154; doi:10.1186/s12864-020-07354-8)

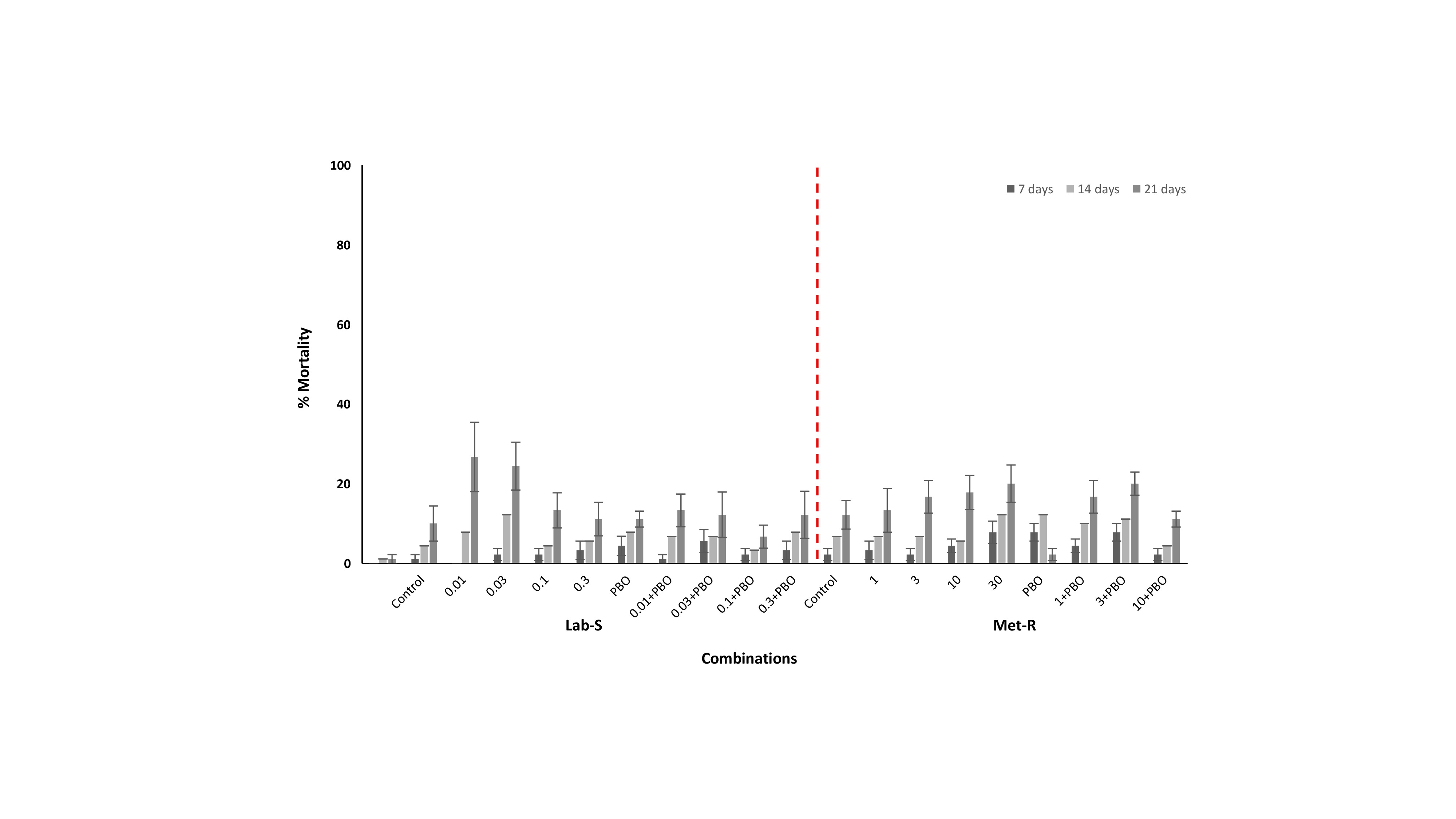

Supplement: Supplementary file 1 — Additional file 1: Fig. S1. Mean mortality (±SEM) of Rhyzopertha dominica after 7, 14 and 21 days of exposure for the susceptible (Lab-S) and resistant (Met-R) strain for all the combinations tested (control, 0.01 mg/kg, 0.03 mg/kg, 0.1 mg/kg, 0.3 mg/kg, PBO, 0.01 mg/kg + PBO, 0.03 mg/kg + PBO, 0.1 mg/kg + PBO, 0.3 mg/kg + PBO for susceptible and control, 1 mg/kg, 3 mg/kg, 10 mg/kg, 30 mg/kg, PBO, 1 mg/kg + PBO, 3 mg/kg + PBO, 10 mg/kg + PBO, 30 mg/kg + PBO for resistant). [file 12864_2020_7354_MOESM1_ESM.jpg]

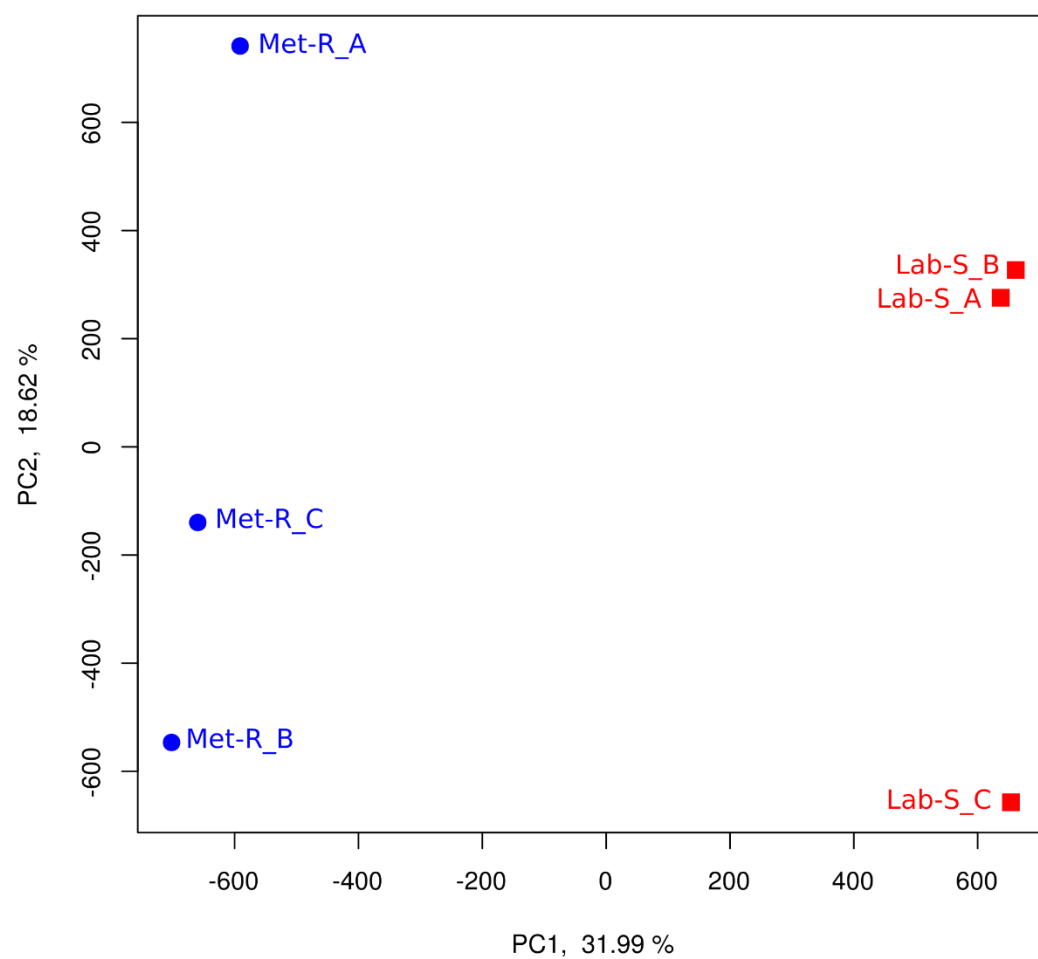

Supplement: Supplementary file 2 — Additional file 2: Fig. S2. Principal components analysis of the transcript expression levels between the resistant (Met-R - blue) and susceptible (Lab-S - red) strains. The two strains are clearly different from each other, a prerequisite for downstream analyses. [file 12864_2020_7354_MOESM2_ESM.pdf]
